# Supplementary figures and images for: Structural and Dynamic Disturbances Revealed by Molecular Dynamics Simulations Predict the Impact on Function of CCT5 Chaperonin Mutations Associated with Rare Severe Distal Neuropathies
Source: Int J Mol Sci. 2023 Jan 19;24(3):2018. doi: 10.3390/ijms24032018 (PMC9917133; doi:10.3390/ijms24032018)

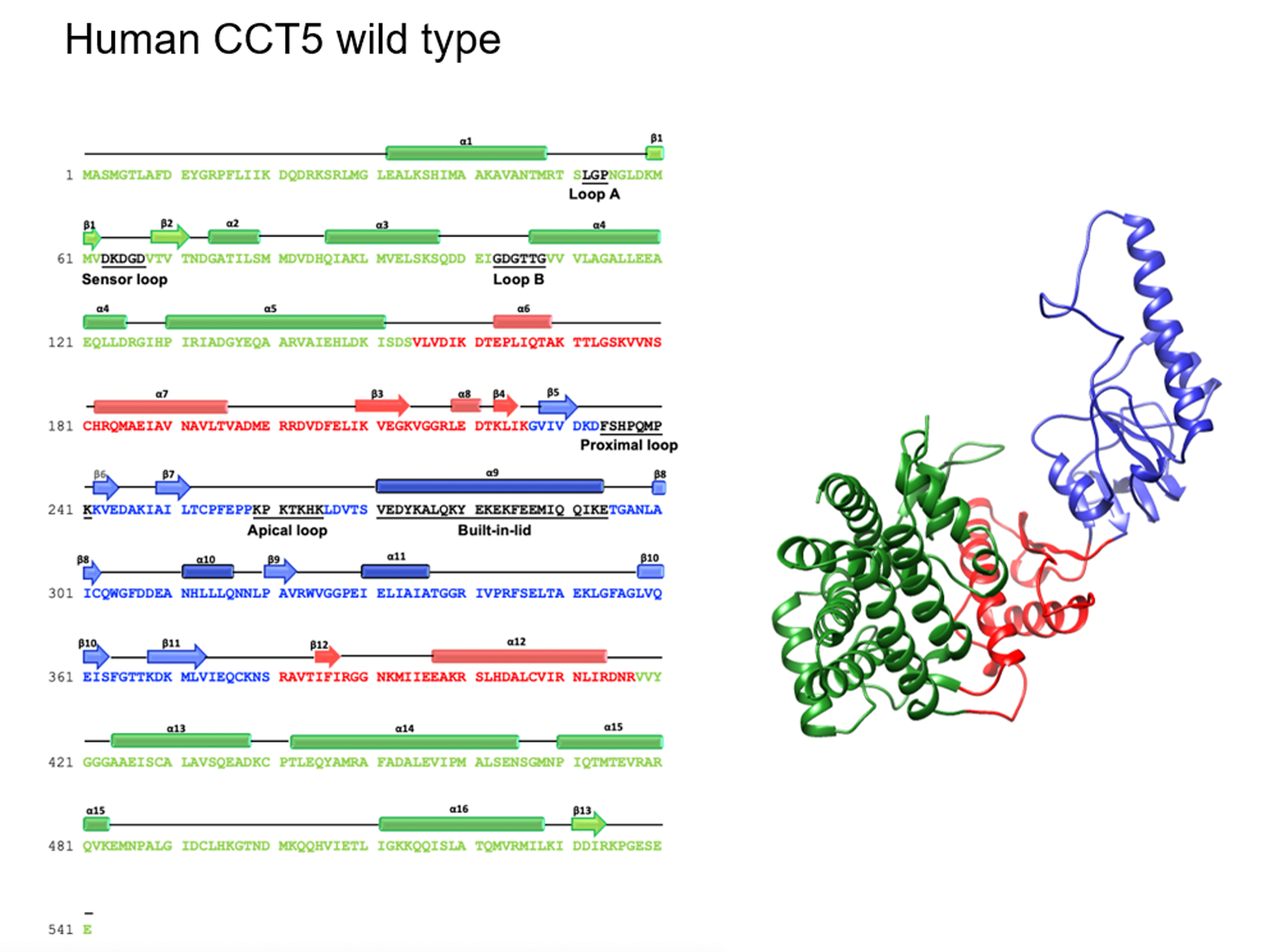

Supplement: Supplementary file 1 [file ijms-24-02018-s001.zip › Figure S1.tif]

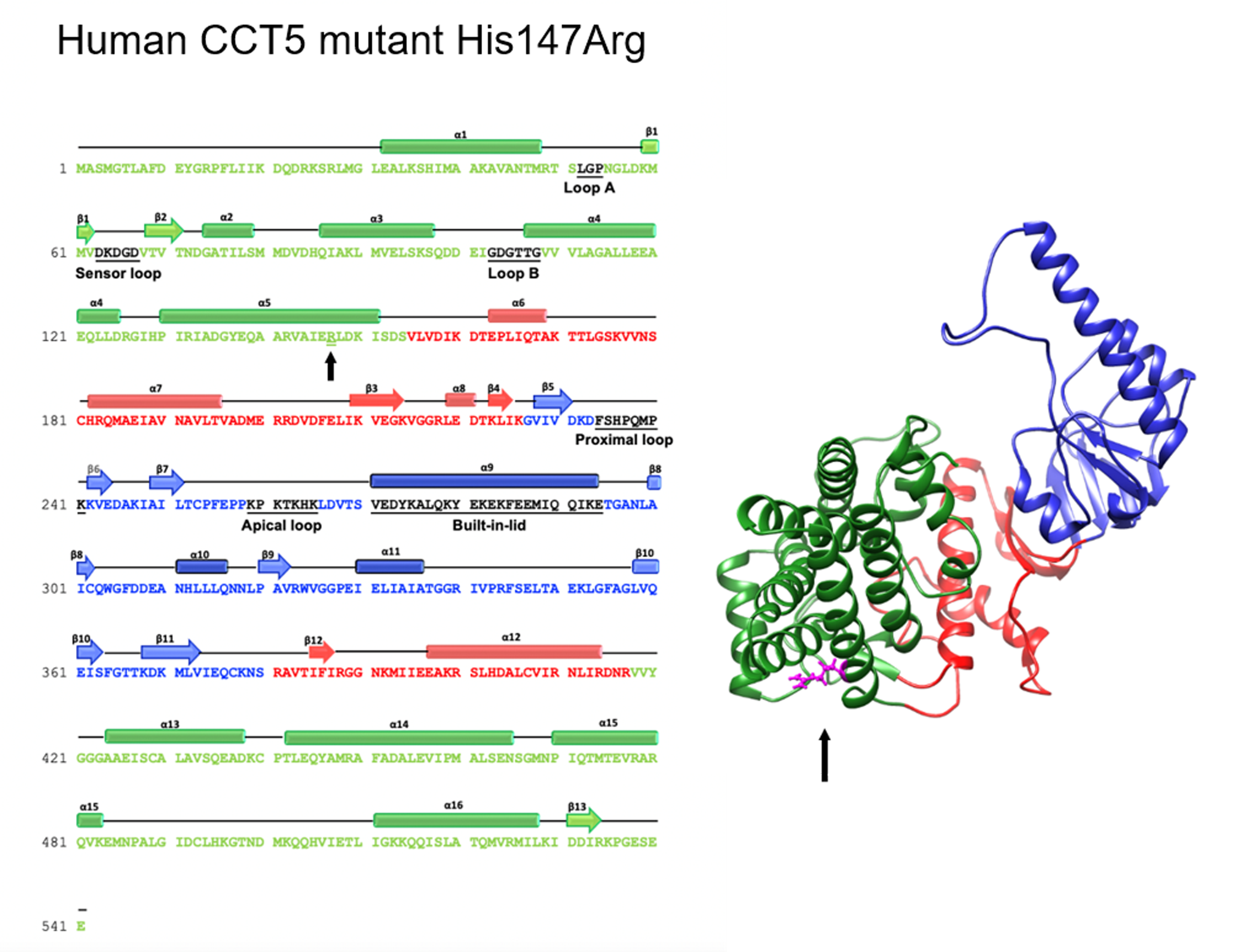

Supplement: Supplementary file 1 [file ijms-24-02018-s001.zip › Figure S2.tif]

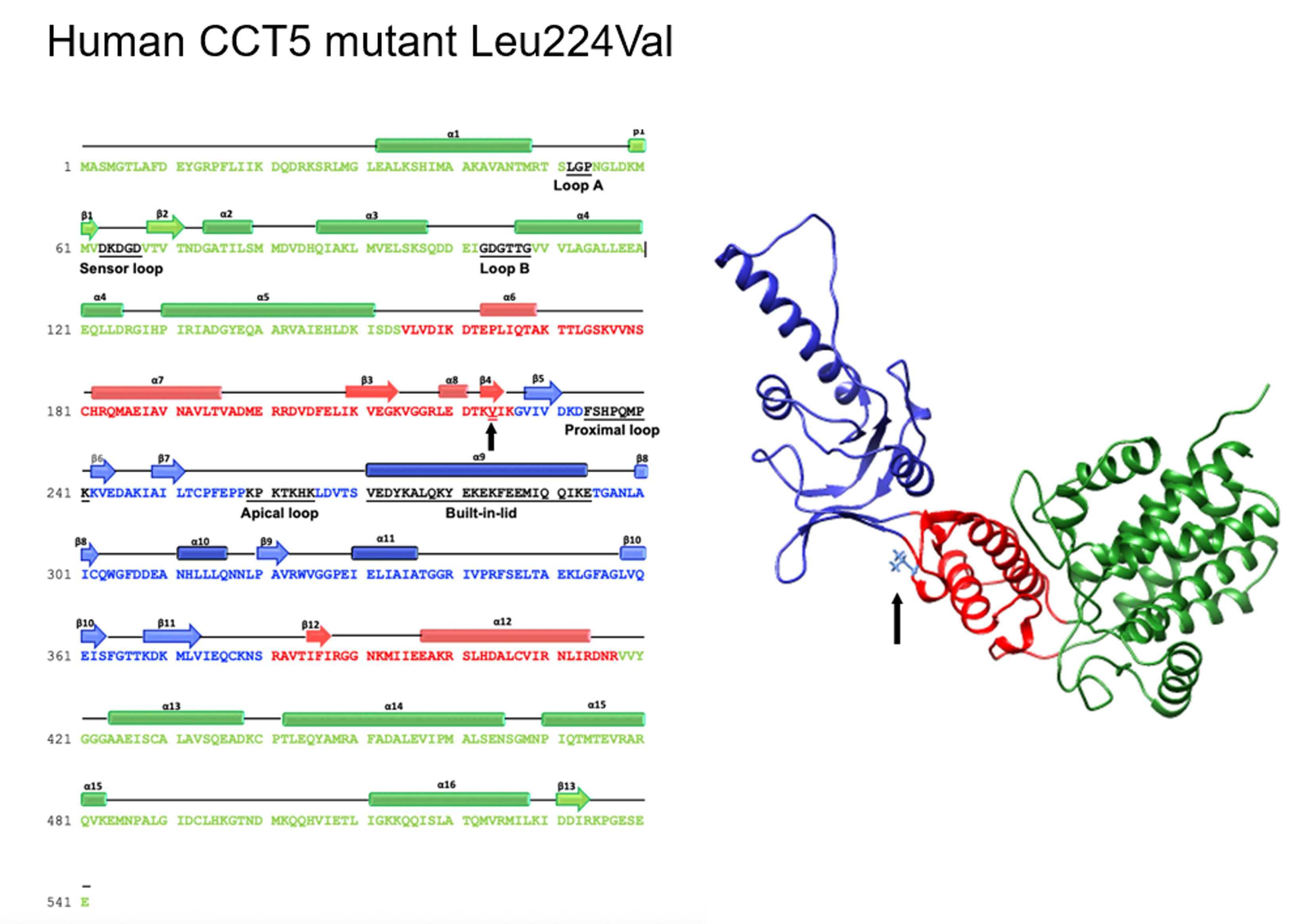

Supplement: Supplementary file 1 [file ijms-24-02018-s001.zip › Figure S3.tif]
